# Whole-Genome Sequence of the Sulfate-Reducing Bacterial Strain SYK, Isolated from a Xenic Culture of an Anaerobic Protist

**DOI:** 10.1128/mra.01257-22

**Published:** 2023-02-27

**Authors:** Ryuji Kondo, Takafumi Kataoka

**Affiliations:** a Department of Marine Science and Technology, Fukui Prefectural University, Obama, Fukui, Japan; Wellesley College

## Abstract

The sulfate-reducing bacterial strain SYK was isolated from a xenic culture of an anaerobic heterolobosean protist which was obtained from a saline lake in Japan. Its draft genome comprises 1 circular chromosome (3,762,062 bp), harboring 3,463 predicted protein- and 65 tRNA-encoding genes and 3 rRNA operons.

## ANNOUNCEMENT

Dissimilatory sulfate-reducing bacteria (SRB) are obligate anaerobic prokaryotes using sulfate as the terminal electron acceptor for anaerobic respiration of various low-molecular-weight fatty acids and hydrogen. SRB are widely distributed in anoxic environments such as aquatic sediments (1). SRB also coexist in the cells of some amitochondriate eukaryotes to scavenge H_2_ produced by hydrogenosomes (2).

A sulfate-reducing bacterium, strain SYK (=DSM 114958, =JCM 35746) was isolated from a xenic culture of an amitochondriate, hydrogenosome-containing heterolobosean protist which was obtained from bottom sediment of the central basin of the saline Lake Hiruga (35°36.212′N, 135°53.349′E) in Fukui, Japan. An aliquot of the protist culture was inoculated in DSMZ 195c medium (https://www.dsmz.de/microorganisms/medium/pdf/DSMZ_Medium195c.pdf) (3), with dl-lactate substituted for l-lactate, and incubated at 20°C in the dark. After several transfers to the same medium, a single colony was isolated using the anaerobic agar-shake tube technique (4). Preliminary phylogenetic analyses based on the 16S rRNA gene sequence suggested that strain SYK is a novel species of the genus *Pseudodesulfovibrio*.

Strain SYK was incubated in a 1-L anaerobic culture bottle (Sanshin) containing 500 mL of DSMZ 195c medium at 20°C in the dark. Then, the cell pellet was harvested by centrifugation at 3,310 × *g* and 4°C for 30 min. The standard phenol-chloroform extraction method was used to extract genomic DNA (gDNA) from the cell pellet, as described previously (5). After the final purification of the extracted DNA using AMPure XP beads (Beckman Coulter), the gDNA was sheared to 10- to 20-kb fragments using a g-TUBE device (Covaris). A long-read DNA library was constructed for strain SYK using the SMRTbell Express template prep kit v2.0 (PacBio), following the manufacturer’s instructions, and sequenced using the PacBio Sequel IIe system. High-fidelity (Hi-Fi) reads were obtained from the subreads, generated using circular consensus sequencing via SMRT Link v10.1.00.119528. The 44,512 HiFi reads containing >3,000 bp were screened using Filtlong v0.2.0 (https://github.com/rrwick/Filtlong), and the remaining 37,532 reads were used for *de novo* assembly with Flye v2.8.3 (6), with polishing times of two, read error of 0.01, minimum overlap length between reads of 10,000, and assembly type meta. Default parameters were used for all software unless otherwise specified. This resulted in a single 3,762,062-bp contig, specified as circular using Flye, with a coverage of 77× (*N*_50_, 3,762,062 bp) and an overall G+C content of 49.4%. The circular contig was rotated to start with the *dnaA* gene using DFAST with the “fix_origin” option (7).

The genome was annotated using DFAST (7), available at the DNA Data Bank of Japan (DDBJ). The draft genome contained 3 rRNA operons, including an exact tandem duplication of the rRNA operon, 65 tRNA genes, and a total of 3,463 coding DNA sequences, including 1,450 genes encoding hypothetical proteins.

### Data availability.

The genome sequence of SYK^T^ has been deposited in the DDBJ under accession number AP026709. The raw reads have been deposited in the DDBJ Sequence Read Archive under accession number DRA014807. The BioProject and BioSample accession numbers are PRJDB14204 and SAMD00521554, respectively.
